# Technical Principles in Elective Surgical Treatment of Left Colon Diverticular Disease: A Scoping Review

**DOI:** 10.3390/jcm14248645

**Published:** 2025-12-05

**Authors:** Luca Emanuele Amodio, Gianluca Rizzo, Federica Marzi, Camilla Marandola, Francesco Ferrara, Vincenzo Tondolo

**Affiliations:** 1 Digestive and Colo-Rectal Surgery Unit, Ospedale Isola Tiberina Gemelli Isola, 00186 Rome, Italy; 2 Unit of General and Oncologic Surgery, Department of Precision Medicine in Medical, Surgical and Critical Care (MePreCC), University of Palermo, “Paolo Giaccone” University Hospital, Via del Vespro 129, 90127 Palermo, Italy

**Keywords:** diverticular disease, sigmoid colectomy, left hemicolectomy, laparoscopy, robotic-assisted surgery, minimally invasive surgery, colorectal surgery

## Abstract

Left colon diverticular disease (LCDD) is prevalent in the aging populations of industrialized countries, with many patients requiring surgery. Elective surgery decisions should consider individual health conditions and quality of life. Typically, surgery is recommended six weeks after an acute episode. This scoping review, following PRISMA-ScR guidelines, analyzed the literature on elective LCDD surgery, focusing on inferior mesenteric artery (IMA) ligation, splenic flexure mobilization (SFM), surgical approach, and extent of resection. The databases searched included PubMed, Embase, and Cochrane Library up to May 2025. Twenty studies met the inclusion criteria: 2 randomized trials (RCTs), 6 systematic reviews, 3 prospective studies, and 9 retrospective cohorts. The findings suggest preserving the IMA and selectively omitting SFM may reduce minor complications without compromising safety. Resection should reach the rectosigmoid junction and include only the affected colon segment. Minimally invasive techniques, especially laparoscopic surgery improve outcomes, reduce morbidity, and are more cost-effective than open surgery. Robotic approaches offer new options for complex cases. Surgical strategies must be tailored to disease severity, patient comorbidities, and anatomy. Further prospective studies are needed to refine guidelines and support personalized surgical decisions in LCDD management.

## 1. Introduction

Colonic diverticular disease (DD) represents one of the most prevalent pathological conditions affecting the lower gastrointestinal tract. The incidence of DD has progressively increased in industrialized countries over the past several decades [1,2]. In Western countries, nearly 50% of individuals over the age of 60 are affected by DD, and approximately 25% of these will experience symptomatic diverticulitis, resulting in an annual hospitalization rate exceeding 750,000 adults per year across Europe [3]. Emergency department admissions and urgent surgical procedures for diverticulitis have significantly decreased over the past two decades [4]. Consequently, elective surgery plays a central role in the management of DD: elective surgical procedures have risen by 38% between 1998 and 2005 (from 15,500 to 21,300 procedures) [5]. Elective surgery has a lower risk of complications and mortality than emergency procedures, with a significantly reduced rate of Hartmann’s procedures, protective stoma creation, and anastomosis leakage (AL) [6,7]. The rate of postoperative complications ranges significantly with the severity of DD. According to Nocera et al., patients operated for uncomplicated diverticulitis or microabscesses have fewer conversions to open surgery (5.0% versus 13.8%), a reduced need for a stoma (0.9% versus 10.7%), and lower rates of major complications (3.8% compared to 10%), when compared to those with macroabscesses, chronic or complicated condition [8]. Indications for elective surgical treatment in DD have changed substantially over time. In the first 2000s, the American Society of Colon and Rectal Surgeons (ASCRS) offered some cautious advice, suggesting that elective colonic resection could be considered after two episodes of uncomplicated diverticulitis or even after just one episode of a more complicated case [5]. The latest clinical practice guidelines from the ASCRS, first updated in 2006 and further revised in 2020, stress the importance of a patient-centered and tailored approach to elective colectomy for DD [9]. Typically, elective surgical resection is advised in case of complications (such as fistulas, bowel obstructions, or strictures) or after effective nonoperative treatment of a diverticular abscess. In case of uncomplicated diverticulitis, it is crucial to personalize the decision, considering the clinical context and individual patient factors (comorbidities and how they affect their overall quality of life (QoL)) [9,10]. In fact, recent studies, including multicenter randomized trials (RCTs) such as DIRECT and LASER, found that laparoscopic (LPS) elective sigmoid resection can significantly enhance the QoL of patients dealing with ongoing abdominal problems, compared to conservative management [11]. The timing of elective surgery is a key factor in the management of DD. Usually, guidelines differentiate between early elective (EE, <6 weeks) and delayed elective (DE, >6 weeks) surgical approaches following initial hospital admission for an acute diverticulitis episode [12]. The DE surgical approach has become standard practice and is now listed among recent guidelines for the management of DD, but no RCT is yet available [12]. A meta-analysis conducted by Vaghir S et al. noticed that the EE group had higher conversion rates, longer operative times, and slower recovery of bowel function compared to the DE group; however, surgical timing did not seem to have a significant impact on major complications or overall mortality. It is important to emphasize that the conversion rate was lowest in the first eight days after the acute episode, which may indicate that the inflammatory response is not as strong during this very early stage. This very early elective approach (until 8 days) can also prevent recurrence, reducing the likelihood of a second hospitalization and improving overall cost-effectiveness [12]. However, colonic evaluation following an episode of complicated diverticulitis is generally recommended to exclude underlying colorectal malignancy. Colonoscopy or CT colonography is typically advised approximately six weeks after the acute episode, to reduce the risk of procedure-related complications—such as perforation—that are more likely during the active inflammatory phase [9,13].

Recurrence rates following elective resection for left colon diverticular disease (LCDD) are estimated in the literature to range between 3% and 13% [14,15,16,17]. The factors that may influence the recurrence have been analyzed thoroughly by Thaler et al. in a 2003 meta-analysis. The only independent factor of recurrence appeared to be the level of anastomosis. Those who had colosigmoid anastomosis were four times more likely to have recurrence when compared to those who had colorectal anastomosis [18]. So, in clinical practice, it is suggested to perform distal colonic resection under the level of the rectosigmoid junction. Over the last twenty years, a wealth of research has explored the long-term results of elective resection for LCDD. With the surgical technique potentially affecting these outcomes, there is an increasing demand for standardized operative management. Traditionally, four main topics have been at the heart of the ongoing discussion in elective surgery for LCDD: choosing between minimally invasive and open approaches, deciding on the vascular ligation method, figuring out how much to mobilize the splenic flexure, and determining the best locations for proximal and distal colonic transection. Currently, no consensus guidelines exist regarding these technical aspects of the surgical procedure. The present comprehensive scoping review aims to fill this gap by systematically mapping and summarizing the existing literature on these technical principles applied in elective colectomy for LCDD.

## 2. Methods

A scoping review is a method for “reconnaissance to clarify working definitions and conceptual boundaries of a topic or field” [19]. Since there is no universal definition of scoping study [20], in this review, we adopted the O’Malley and Arksey methodological framework: identifying the research question, searching for relevant studies, selecting studies, charting the data, collating, summarizing, and reporting the results [21]. A comprehensive literature search was conducted in PubMed, Embase, and the Cochrane Library from database inception to May 2025, following the PRISMA-ScR extension. The search strategy was developed iteratively, consistent with the methodological framework proposed by Arksey & O’Malley, to ensure broad coverage of all studies addressing technical principles in elective colectomy for left colon diverticular disease (LCDD). Search terms combined controlled vocabulary (MeSH/EMTREE) and free-text words related to DD, elective surgery, colectomy, surgical technique, inferior mesenteric artery (IMA) ligation, splenic flexure mobilization (SFM), surgical approach (LPS, robotic (RBT), open), and extent of resection. No restrictions were applied regarding study design or publication date. Only studies involving adults undergoing elective surgery for LCDD were included. The full electronic search strings and the eligibility criteria are reported in Supplementary Materials S1 and Supplementary Materials S2, respectively. Two reviewers independently screened titles and abstracts, followed by full-text evaluation of potentially eligible studies. Disagreements were resolved by consensus. Reasons for exclusion were documented in the PRISMA-ScR flow chart. Data extraction followed a structured, predefined form and included study characteristics (author, year, country, design), sample size, technical principle investigated (IMA ligation, SFM, extent of resection, surgical approach), and main outcomes. Data were charted by one reviewer and cross-checked by a second reviewer. Given the heterogeneity of study designs and outcomes, results were summarized descriptively, and no meta-analysis or quantitative synthesis was performed. This scoping review was not prospectively registered on a public registry, like PROSPERO or the Open Science Framework.

## 3. Results

**Figure 1:**
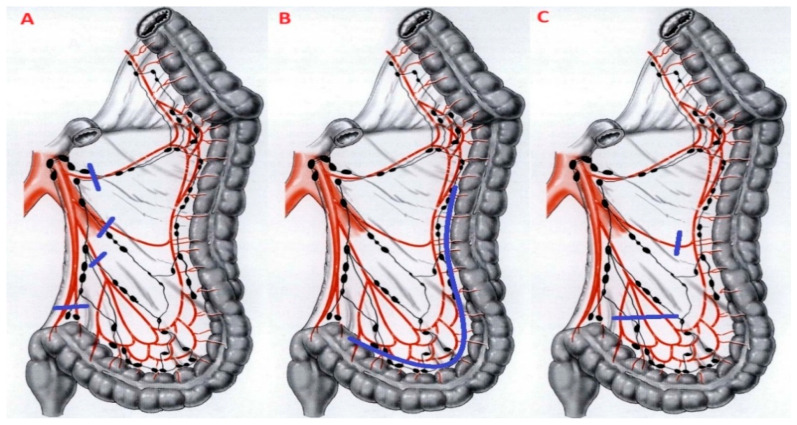
Surgical techniques used for IMA preservation in left colon resection. Blue bracket lines indicate the level of vascular ligation. Image (**A**): Valdoni technique; image (**B**): tubular resection technique; image (**C**): peripheral dissection technique.

The PRISMA-ScR checklist (Preferred Reporting Items for Systematic reviews and Meta-Analyses extension for Scoping Reviews) was followed as SDC. PRISMA flow chart is shown in Figure 1 [22]. The initial search produced 137 potentially relevant articles. A total of 59 articles were screened for relevance of titles and abstracts; 22 articles were further fully evaluated for eligibility, and 2 were excluded. Thus, 20 studies were finally included in the review [16,18,23,24,25,26,27,28,29,30,31,32,33,34,35,36,37,38,39,40]. Twenty studies met the inclusion criteria: 2 RCTs, 6 systematic reviews, 3 prospective studies, and 9 retrospective cohorts. Study characteristics are reported in Table 1. The main results are summarized in Table 2.

### 3.1. Inferior Mesenteric Artery (IMA) Ligation

Tocchi et al. (2001) conducted an RCT including 163 patients undergoing left colectomy for complicated LCDD, comparing outcomes of IMA preservation through skeletonization (or low IMA ligation) versus IMA ligation at its origin (or high IMA ligation). Radiological and clinical AL were significantly higher when the IMA was ligated (18.1% vs. 7%, *p* = 0.02; 10.4% vs. 2.3%, *p* = 0.03, respectively), supporting the preservation of the natural rectal blood supply, which helps reduce AL risk [23]. Lehmann et al. (2011) retrospectively analyzed 130 colectomies for LCDD and found no significant difference in AL rates when comparing patients in whom IMA was preserved to those in whom it was sacrificed [24]. Cirocchi et al. (2012) conducted a systematic review comparing high and low IMA ligation in elective surgery for LCDD and found no differences in AL, morbidity, or mortality between the two approaches [25]. Mari et al. (2017) prospectively compared 66 patients undergoing LPS colectomy for LCDD with low IMA ligation versus IMA preservation. They found no differences in major complications, bowel function, or genitourinary outcomes; however, operative time was shorter (135 ± 28 vs. 175 ± 31 min, *p* < 0.05) with lower blood loss (55 ± 16 vs. 81 ± 22 mL, *p* < 0.05). They concluded that both approaches were safe and functionally equivalent [26]. Jolivet et al. (2020) prospectively evaluated 25 consecutive male patients undergoing LPS resection for LCDD with high IMA ligation and found no significant differences between preoperative and 6-month postoperative bowel (Jorge–Wexner score), urinary (IPSS), or sexual function (IIEF-5 score), while QoL significantly improved in terms of general health and medical status, supporting the safety of high-tie ligation without functional impairment [27]. De Nardi et al. (2018) [28] reviewed 219 elective LPS colectomies for LCDD, and the mean operative time was 225 ± 43.4 min in the high IMA ligation group and 191 ± 41.7 min in the IMA preservation group (*p* = 0.002). No differences were observed in the rate of overall complications, stoma formation, restoration of bowel function, and post-operative LOS [28].

Cirocchi et al. (2012) [25] conducted a systematic review and meta-analysis including 12 studies and 2812 patients undergoing colectomy for LCDD (1612 with IMA ligation vs. 1200 with preservation). IMA preservation was associated with longer operative time (MD 21.96, CI 4.52–39.39, *p* = 0.0005) and a higher conversion rate to open surgery (7.6% vs. 4.3%, *p* = 0.02) [29]. Silvestri et al. (2023) performed a prospective non-randomized controlled trial on 122 elective colectomies for LCDD and demonstrated significantly fewer postoperative defecatory disorders in the preservation group (Wexner incontinence score 1.06 ± 1.63 vs. 3.88 ± 3.11, *p* < 0.001), as well as fewer urinary symptoms (IPSS 0.73 ± 1.85 vs. 4.33 ± 4.69, *p* < 0.001) and better global QoL scores (*p* = 0.02), confirming a functional advantage for IMA preservation without increasing complications [30]. Agnesi et al. (2024) [31] conducted a systematic review including 989 patients from 11 studies comparing three IMA-preserving techniques: group A (Valdoni technique), Group B (tubular resection technique), and Group C (peripheral dissection technique). The statistically significant results are reported below. Patients in Group A experienced longer operative times (174.5 ± 27.4 min) and hospital stays (11.4 ± 3.6 days) compared to Groups B and C (165.9 min and 152.35 ± 46.9 min; 8.4 ± 5.7 days and 8.3 ± 3.6 days, respectively). Group A was associated with higher rates of AL (5%) compared to Group C (1.1%) and a higher incidence of bleeding (13%) compared to Group B (1.8%). The authors concluded that peripheral dissection and tubular resection provide the optimal balance of safety and efficiency for elective diverticular resections [31]. Mari et al. (2025) [32] performed a monocentric RCT including 219 patients undergoing elective LPS resection for LCDD. The occurrence of defecatory disorders and their impact on QoL were assessed with 6 questionnaires, administered at 6 and 12 months and after 6 years from surgery, and with an anorectal manometry performed after 6 months and 5 years. They observed a significantly lower incidence of postoperative defecatory disorders in the IMA-preservation group and superior long-term QoL scores. No differences were reported in overall morbidity or AL, confirming that IMA preservation improves function without compromising safety [32].

### 3.2. Splenic Flexure Mobilization (SFM)

Schlussel et al. (2017) [33] retrospectively analyzed 208 elective colectomies for LCDD and found that SFM was performed in 54% of cases. SFM significantly increased operative time (226 min vs. 180 min, *p* < 0.01) and seemed to be linked to higher rates of minor complications (OR 2.8, *p* = 0.05) without leading to higher major morbidity or organ-space infection rates (*p* = 0.34), thus suggesting a more selective rather than routine adoption [33]. Barraud et al. (2024) [34] conducted a multicenter French retrospective cohort study including 1014 elective colectomies for LCDD (676 with SFM versus 338 without SFM). They performed a propensity score analysis showing that the omission of SFM was not significantly associated with an increased cumulative incidence of severe postoperative complications within 90 days after surgery. Furthermore, the overall rate of morbidities, including AL, hemorrhage, and reintervention, was not negatively influenced by choosing not to perform SFM. The authors concluded by supporting a selective rather than routine indication [34].

### 3.3. Extent of Resection and Anastomotic Level

Parks (1969) [35] described the natural history of DD in 521 patients, showing that progression usually occurred within the initially affected segments, most often the sigmoid colon (96%), while extension to other colonic regions was rare. These findings suggested that recurrence after surgery is primarily related to residual disease in the sigmoid rather than proximal extension [35]. Wolff et al. (1984) followed 61 patients after elective resection for LCDD and found that only 11.4% developed recurrent diverticulitis and 14.7% had radiologic progression, with minimal new diverticula formation; recurrence occurred in 7 patients, and none required reoperation, supporting limited sigmoid resection rather than extended colectomy [36]. Benn et al. (1986) [16] reviewed 501 patients who underwent resection for LCDD and observed a significantly higher recurrence rate when the distal anastomosis involved the sigmoid colon compared with the rectum (12.5% vs. 6.7%, *p* < 0.05), with recurrence occurring between 1 and 139 months (median 45 months). There were no statistically different rates of reoperation [16]. Thaler et al. (2003) [18] analyzed 236 patients after elective colectomy for LCDD. A logistic regression of the following variables was undertaken: patient demographics, duration of preoperative symptoms, previous admissions and abdominal surgery, surgical approach (LPS or open), postoperative complications, SFM, anastomotic technique (handsewn or stapled), specimen length, inflammation at proximal resection margin, and anastomotic level (colosigmoid or colorectal). They identified the anastomotic level as the only independent predictor of recurrence: colosigmoid anastomosis increased recurrence fourfold compared with colorectal anastomosis (12.5% vs. 2.8%, OR 1.12–14.96; *p* = 0.033) [18].

### 3.4. Surgical Approach (Laparoscopic (LPS), Robotic (RBT), Open)

Masoomi et al. (2011) [37] analyzed a large national database of elective colectomies for LCDD, including 124,734 patients (14,562 in the LPS group). The overall intraoperative complication rate was significantly lower in the LPS group (0.63% vs. 1.15%, *p* < 0.01). All evaluated postoperative complications (ileus, abdominal abscess, AL, wound infection, bowel obstruction, urinary tract infection, pneumonia, respiratory failure, venous thromboembolism) were significantly higher for the open procedures. Also, mortality was four times higher in the open group (open, 0.54%; LPS, 0.13%, *p* < 0.01). The LPS group had a shorter mean hospital stay (LPS, 5.06 days; open, 6.68 days, *p* < 0.01) and lower total hospital charges (LPS, $36,389; open, $39,406, *p* < 0.01) than the open group. They concluded that elective LPS surgery is safe and can be considered the preferred operative option [37].

Gaertner et al. (2013) carried out a systematic review including nine studies and 931 patients, which reported that elective LPS colectomy for LCDD is associated with increased operative time, decreased postoperative pain, fewer postoperative complications, lower rates of paralytic ileus, and shorter length of hospital stay (LOS) compared to open colectomy [38]. Giuliani et al. (2021) conducted a meta-analysis of nine studies involving 4177 patients and found out that patients undergoing colectomy with LPS approach had a significantly higher risk of conversion into an open procedure when compared to those undergoing RBT colectomy (12.5% vs. 7.4%, *p* < 0.00001) and shorter LOS (SMD−0.21 [−0.32,−0.11], Z = 3.94, *p* < 0.0001, I2 = 45%), with a longer operating time (SMD of 0.49 [0.39, 0.60]; *p* < 0.00001, I2 = 94%) [39]. Larkins et al. (2022) [40] confirmed these findings in a systematic review and meta-analysis. Fifteen articles (8 cohort studies and 7 case series) reporting 3711 RBT resections for LCDD were analyzed. RBT colectomy had a lower conversion rate than LPS colectomy (OR: 0.57; 95% CI: 0.49–0.66, *p* < 0.001), comparable morbidity and mortality, but longer operative time (Hedge’s G: 0.43; 95% CI: 0.04–0.81, *p* = 0.03). They concluded that RBT surgery is safe and may allow resection of DD through a minimally invasive approach even in challenging conditions [40].

## 4. Discussion

This scoping review mapped current evidence on four key technical principles in elective colectomy for LCDD. Strengths of this review include adherence to PRISMA-ScR methodology, comprehensive literature coverage, and systematic charting of technical aspects. Limitations include the absence of protocol registration, heterogeneity of included studies, and lack of formal critical appraisal, which is consistent with the scope of this review. Differences in methodology, patient selection, and operative approaches limit the comparability of results and preclude quantitative meta-analysis. Consequently, the interpretation of outcomes should be approached with caution, and conclusions should be considered in the context of this variability.

### 4.1. Inferior Mesenteric Artery (IMA) Ligation

The optimal level of IMA ligation remains a controversial topic. While high IMA ligation (at its origin) has been historically preferred for oncologic resections, in benign disease, it may not be necessary. In recent years, numerous scientific investigations have examined the correlation between the degree of IMA ligation and intraoperative results, postoperative and functional outcomes. As for intraoperative outcomes, IMA’s preservation seems to be associated with a higher conversion rate and longer operative times, with a comparable bleeding rate. This can be attributed to the fact that distal ligation involves intramesocolic dissection instead of a straightforward dissection along avascular planes. In those situations, in which mesocolon is chronically inflamed, thickened, or fibrotic—especially in the case of intramesocolic abscess—the chance of misidentifying anatomical landmarks rises, thus contributing to a higher conversion rate. However, it is important to evaluate these results with caution: it is unclear when the conversion took place (before, during, or after the vascular preparation), and conversion rates are significantly influenced by the surgeon’s technical skill and experience [29]. The ASCRS suggests that a mid-mesenteric dissection with preservation of the IMA may decrease the incidence of the AL, as suggested by Tocchi et al. [23] in their RCT [9]. Although no metal analysis has demonstrated a statistically significant advantage for this approach, the topic remains controversial [24,25,26,27,28]. However, it should be noted that different techniques of peripheral IMA ligation exist. Only the study conducted by Agnesi et al. in 2024 specifically analyzed the three different techniques of peripheral IMA ligation [31]. They distinguished: the Valdoni technique, which involves the skeletonization of the IMA with subsequent division of all branches directed towards the left and sigmoid colon [42]; tubular resection technique, first described by Gall in 1982 [43], with vascular control achieved near the colonic wall [44]; the peripheral dissection with division at the intramesocolic level of the distal branches of the sigmoid vessels [26] (Figure 1). Agnesi et al. (2024) performed a systematic review and demonstrated that Valdoni’s dissection was associated with higher AL and bleeding rates and longer operative times compared with both tubular resection and peripheral dissection [31]. The most recent and comprehensive systematic review on functional outcomes was published by Cirocchi et al. in 2022 [29]. Twelve studies were included in the meta-analysis: 3 RCTs and 9 non-RCTs, a total of 2812 patients (1612 underwent IMA ligation versus 1200 with IMA preservation). They demonstrated that the level of IMA ligation did not influence functional outcomes—bowel, urinary, or sexual function—and consequently had no impact on overall QoL [29]. These results are quite counterintuitive: one would expect the risk of injuring the superior hypogastric plexus and hypogastric nerves if lower dissection is performed far from the aortic plane, as in the case of distal IMA ligation. Therefore, we would have expected better genitourinary and bowel functional outcomes with this approach [41]. Furthermore, distal IMA ligation spares the hypogastric nerve components, which accompany the IMA down to the rectum and distal sigmoid, potentially ensuring better bowel function [45]. Subsequently, two prospective trials, one of which was randomized, including a total of 341 patients, demonstrated improved bowel and genitourinary functional outcomes in cases of low IMA ligation [30,32]. In the absence of definitive scientific evidence, an implicit principle continues to guide surgical decision-making regarding QoL: “*if you cannot improve it, at least do not worsen it.*” [46]. This inferential perspective aligns with the 2020 recommendations of the European Society of Coloproctology (ESCP), which advised preserving the IMA “in cases where there is no suspicion of cancer to optimize the preservation of the vascularization and of the autonomic nerves” [47].

### 4.2. Splenic Flexure Mobilization (SFM)

Although SFM is commonly performed to allow a tension-free anastomosis in left-sided colectomies, its routine application in the surgical treatment of LCDD is controversial. In case of a redundant sigmoid colon, the amount of resected colon may not require the need for SFM. Furthermore, SFM is a technically challenging step of a complex operation: LPS performance of this technique requires additional skills and experience in minimally invasive surgery [33]. In line with this, newer European guidelines propose a conditional recommendation (grade C) that performing SFM is left to the operating surgeon’s judgment [47]. In the oncological setting of colorectal cancer (left hemicolectomy or rectal resection), several studies have demonstrated minimal to no difference in postoperative outcomes and overall survival between patients undergoing SFM and those in whom SFM was not performed [48,49]. Different studies have also analyzed this aspect in LCDD. Schlussel AT et al., in a single-center retrospective cohort study of 208 patients (between 2007 and 2015), investigated for the first time the surgical role of SFM in patients treated with LCDD. They noticed that the SFM group had significantly longer operative times and a higher rate of minor postoperative complications (superficial or deep surgical site infection, pneumonia, unplanned intubation, urinary tract infection, or deep vein thrombosis). There was no significant difference in the risk of major postoperative adverse events (including organ space infection) between the two groups, and there was no recorded iatrogenic injury of the spleen [33]. In 2024, a trial by Barraud A et al. analyzed 4398 patients who underwent colectomy for LCDD, implementing two distinct propensity score matching techniques to minimize selection bias. This study confirmed that SFM’s omission significantly reduced operative time and minor complications, with no significant differences in the rates of bleeding complications between SFM and non-SFM groups [34].

### 4.3. The Extent of Colonic Resection

Although a great deal of research has been conducted on the natural history of DD since the 1980s, there is still widespread disagreement among clinicians regarding the optimal extent of colonic resection. According to Parks et al. DD tends to progress predominantly within the segments initially affected, with an unpredictable spreading pattern to adjacent colonic regions. Proximal extension of the disease is rare after surgical resection, and a second resection is uncommon [35]. According to other authors, a limited left colonic resection is typically sufficient, and it is not necessary to resect the whole colon with diverticula. In fact, diverticulitis affects almost exclusively the sigmoid colon, and patients with pancolonic DD have no worse prognosis. Typically, 10 to 20 cm of sigmoid can be identified and removed when the inflammatory process has sufficiently tapered off [36]. Another aspect to consider when determining the extent of resection was suggested by Benn et al. in 1986 [16]. They concluded that avoiding retention of a distal sigmoid cuff minimizes the risk of inflammation recurrence, particularly given that diverticula anatomically do not arise in the rectum [16]. In particular, Thaler K et al. demonstrated that distal resection involving the sigmoid colon is associated with a fourfold increased risk of recurrence compared to distal resection at the rectosigmoidal junction [18]. Therefore, in clinical practice, it is advisable to perform a resection of the segment of the left colon involved in diverticular inflammation, extending distally to the level of the rectosigmoid junction.

### 4.4. Surgical Approach (Laparoscopic (LPS), Robotic (RBT), Open)

In the past decades, the treatment of LCDD has immensely benefited from the emergence of LPS surgery. Although it faced initial resistance due to technical difficulty and complication risks, the LPS approach has proven to be effective and safe. The refinement of LPS surgical instruments improved surgical technique, and an increasing number of trials supported the use of LPS in comparison to open surgery [38]. Multiple systematic reviews have compared intraoperative outcomes of LPS and open colectomy for LCDD. The more comprehensive and recent one is by Gaertner et al. (2013) [38]. LPS colectomy generally involves a longer operative time compared to the open approach. However, this disadvantage is counterbalanced by significantly reduced intraoperative blood loss and lower physiological stress on the patient during the procedure [38]. Outcomes following surgery have also been the focus of careful examination. The systematic review of Masoomi et al. in 2011 [37] provided a definitive advantage toward the LPS approach. Specifically, all postoperative complications examined (which included ileus, intra-abdominal abscess, AL, wound infection, bowel obstruction, urinary tract infection, pneumonia, respiratory failure, and venous thromboembolism) were significantly more frequent in patients treated with the open approach. In contrast, LPS was characterized not only by a lower incidence of these complications but also by lower postoperative mortality, shorter hospital stays, and reduced overall healthcare costs. These findings highlight the advantages of minimally invasive surgery in enhancing patient recovery and optimizing resource utilization in elective colectomy for LCDD [37]. In this regard, a recent retrospective series by Mari et al. [32] confirmed that surgical elective treatment of LCDD is safe and effective, with low rates of complications and a notable long-term improvement in QoL, when carried out using a standardized LPS technique [50]. The RBT-assisted surgery is also increasingly gaining attention due to its enhanced precision and ergonomic advantages. In 2022, Larkins et al. published a systematic review and meta-analysis of 15 studies, which included 3711 RBT resections for LCDD. Their findings indicate that an RBT approach has a lower conversion rate, without effects on the rate of major perioperative complications, which remains comparable to other techniques [40]. These outcomes had already been described by Giuliani et al. in a meta-analysis published in 2021, which highlighted the reduction in conversion rate and its impact on LOS and overall costs [39]. This lower conversion rate may be explained by the RBT platform’s ability to allow minimally invasive surgery in difficult cases. RBT approach effectively extends the limits of what is feasible through a minimally invasive approach. Although RBT surgery is characterized by longer operating times, they are comparable to those of open and LPS approaches. With continuous technological advancements, including RBT staples, improved multi-quadrant access, and enhanced docking efficiency, emerging data are expected to demonstrate a reduction in operative time [40].

## 5. Conclusions

**Figure 2:**
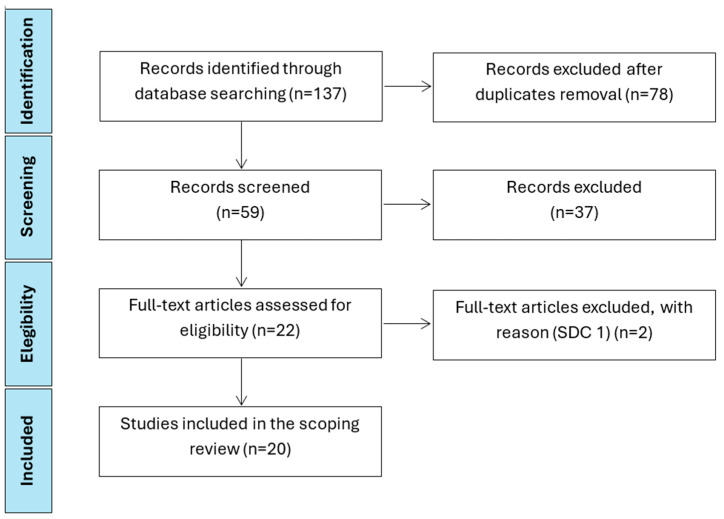
PRISMA flow diagram of study research.

The surgical management of LCDD has evolved significantly. Careful identification and preservation of the hypogastric nerve and plexus are mandatory. Although a recent prospective study showed better functional outcomes with high ligation, previous reviews did not reveal significant differences, so the evidence regarding the ideal level of IMA ligation is still conflicting. Additionally, high IMA ligation appeared to be associated with a more complex and hemorrhagic inframesocolic dissection. This remains a topical area of interest, and further RCTs will be helpful in elucidating its functional impact. Even if avoiding SFM should reduce the operative time, the routine SFM does not seem to be associated with an increasing intraoperative complication rate, but it surely guarantees an objective tension-free anastomosis. The extent of colonic resection should be limited to the area of complicated DD (also according to vascular perfusion), but the distal resection margin should be located at the upper rectum in an area surely free of diverticula. Moreover, the standardization of left hemicolectomy for LCDD, using the same skills of oncological resection, should be able to improve the learning curve of young surgeons, potentially improving the postoperative outcomes. Since the advent of minimal invasive surgery, LPS colectomy is now the gold standard due to its superior postoperative outcomes compared to the open approach. Although RBT surgery shows promise in lowering conversion rates, especially in complicated cases, its current application in the treatment of LCDD is still constrained by its high cost and learning curve. As a result, its adoption is still evolving and not yet routine. New prospective trials are needed to clarify the true benefit of the RBT approach in the surgical management of LCDD. In conclusion, elective surgical management of LCDD should be individualized—balancing timing, technical aspects, and anatomical features—while adhering to standardized operative principles. Specifically, colonic resection should be limited to the segment affected by diverticular inflammation and extended up to the rectosigmoid junction. SFM should be performed when anatomically necessary to ensure a tension-free anastomosis. A minimally invasive approach—LPS or RBT—should be preferred, depending on availability and the surgeon’s expertise. Future prospective trials are needed to better define long-term outcomes and to individualize evidence-based clinical practice guidelines, particularly regarding the RBT approach and the optimal level of IMA ligation (Figure 2).

## Figures and Tables

**Table 1:** Included studies. RCT: Randomized Controlled Trial; SFM: Splenic Flexure Mobilization; IMA: Inferior Mesenteric Artery; LPS: Laparoscopic; RBT: Robotic.

Author (Year)	Study Type	N. Patients	Technical Principles
Agnesi S et al. (2024) [31]	Systematic review	989	IMA preservation
Barraud et al. (2024) [34]	Multicentric retrospective cohort	1014	SFM
Benn et al. (1986) [16]	Retrospective cohort	501	Extent of resection and anastomotic level
Cirocchi et al. (2012) [25]	Systematic review and meta-analysis	400	IMA preservation vs. ligation
Cirocchi et al. (2022) [41]	Systematic review	2812	IMA preservation vs. ligation
De Nardi et al. (2018) [28]	Retrospective cohort	219	IMA preservation vs. ligation
Gaertner et al. (2013) [38]	Systematic review	931	Surgical approach: LPS vs. open
Giuliani G et al. (2022) [39]	Systematic review and meta-analysis	4177	Surgical approach: LPS vs. RBT
Jolivet et al. (2020) [27]	Prospective non-randomized controlled trial	52	IMA preservation
Larkins et al. (2022) [40]	Systematic review and meta-analysis	3711	Surgical approach: LPS vs. RBT
Lehmann et al. (2011) [24]	Retrospective cohort	130	IMA preservation vs. ligation
Mari et al. (2017) [26]	Prospective multicenter parallel study	66	IMA preservation vs. ligation
Mari et al. (2025) [32]	RCT	219	IMA preservation vs. ligation
Masoomi et al. (2011) [37]	Retrospective cohort	124,734	Surgical approach: LPS vs. open
Parks (1969) [35]	Retrospective cohort	521	Extent of resection and natural history
Schlussel et al. (2017) [33]	Retrospective cohort	208	SFM
Silvestri et al. (2023) [30]	Prospective non-randomized controlled trial	122	IMA ligation vs. preservation
Thaler et al. (2003) [18]	Retrospective cohort	236	Extent of resection
Tocchi A et al. (2001) [23]	RCT	163	IMA preservation vs. ligation
Wolff et al. (1984) [36]	Retrospective cohort	61	Extent of resection

**Table 2:** Main results. IMA: Inferior Mesenteric Artery; AL: Anastomotic Leak; QoL: Quality of Life; SFM: Splenic Flexure Mobilization; LPS: Laparosocpic; RBT: Robotic; LOS: Length of Hospital Stay.

Technical Principles	Main Results
IMA preservation	Higher conversion rate and longer operative times, with comparable bleeding rate. Lower AL rate. Comparable functional outcomes and overall QoL.
SFM	Longer operative times and comparable bleeding rate. Higher rate of minor postoperative complications, comparable rate of major postoperative complications. To perform according to operating surgeon’s judgment.
The extent of colonic resection	Resection of the segment of the left colon involved diverticular inflammation, extending distally to the level of the rectosigmoid junction.
Surgical approach	LPS vs. Open: longer operative time, reduced intraoperative blood loss and lower physiological stress. Lower incidence of postoperative complications, lower postoperative mortality, shorter hospital stays and reduced overall healthcare costs RBT vs. LPS: longer operative time, lower conversion rate. Shorter LOS and lower overall costs

## Data Availability

No new data were created or analyzed in this study.

## Supplementary Materials

The following supporting information can be downloaded at: https://www.mdpi.com/article/10.3390/jcm14248645/s1, Supplementary Materials S1. Full electronic search strings; Supplementary Materials S2. Eligibility criteria.
