# Supplementary material for: Technical Principles in Elective Surgical Treatment of Left Colon Diverticular Disease: A Scoping Review
Source: J Clin Med. 2025 Dec 5;14(24):8645. doi: 10.3390/jcm14248645 (PMC12733411; doi:10.3390/jcm14248645)
Supplement: Supplementary file 1 [file jcm-14-08645-s001.zip › jcm-3997137-supplementary.pdf]

Supplementary Materials S1**.** Full electronic search strings.

PubMed search strategy

("Diverticulitis, Colonic"[Mesh] OR "Diverticulosis, Colonic"[Mesh]

OR diverticular disease OR diverticulitis OR diverticulosis

OR sigmoid diverticulitis OR left colon diverticulitis OR LCDD)

AND

(elective OR elective surgery OR elective colectomy OR planned surgery)

AND

(colectomy[Mesh] OR sigmoidectomy OR left colectomy

OR sigmoid resection OR colorectal resection)

AND

(technique OR technical OR surgical technique OR surgical approach

OR laparoscopic OR laparoscopy OR robotic OR robotic-assisted OR open surgery

OR inferior mesenteric artery OR IMA ligation OR high tie OR low tie

OR splenic flexure mobilization OR splenic flexure

OR extent of resection OR anastomosis level OR anastomotic level)

Embase search strategy

('diverticular disease'/exp OR 'diverticulitis'/exp

OR diverticulitis:ti,ab,kw OR diverticulosis:ti,ab,kw

OR 'left colon' AND (diverticulitis OR diverticular))

AND

(elective:ti,ab,kw OR 'elective surgery'/exp OR 'elective colectomy':ti,ab,kw)

AND

('colectomy'/exp OR colectomy:ti,ab,kw

OR sigmoidectomy:ti,ab,kw OR 'left colectomy':ti,ab,kw)

AND

('surgical technique'/exp OR 'surgical approach'/exp

OR technique:ti,ab,kw OR laparoscopic:ti,ab,kw OR laparoscopy/exp

OR robotic:ti,ab,kw OR open:ti,ab,kw

OR 'inferior mesenteric artery'/exp OR 'IMA ligation':ti,ab,kw

OR 'high tie':ti,ab,kw OR 'low tie':ti,ab,kw

OR 'splenic flexure'/exp OR 'splenic flexure mobilization':ti,ab,kw

OR 'extent of resection':ti,ab,kw OR 'anastomotic level':ti,ab,kw)

Cochrane Library search strategy

(diverticulitis OR diverticular disease OR diverticulosis)

AND

elective surgery OR elective colectomy)

AND

(colectomy OR sigmoidectomy OR left colectomy)

AND

(IMA OR inferior mesenteric artery OR high tie OR low tie

OR splenic flexure OR anastomosis OR laparoscopic OR robotic OR open)

Supplementary Materials S2**.** Eligibility criteria

Inclusion criteria:

- Adult patients (≥18 years).
- Elective surgical treatment for left colon diverticular disease.
- Studies reporting at least one technical principle:
    - Inferior mesenteric artery (IMA) ligation level,
    - Splenic flexure mobilization (SFM),
    - Surgical approach (laparoscopic, robotic, open),
    - Extent of resection and anastomotic level.
- Any study design (RCTs, cohort studies, case series, systematic reviews, meta-analyses).

- Full-text available in English, Italian, French, Spanish or German.

Exclusion criteria:

- Emergency surgery.
- Pediatric populations.
- Case reports.
- Studies not addressing at least one of the predefined technical principles.
- Non-original data (letters, expert opinions without data).
